# The Treatment of Infected Root-Canals with Kalium and Natrium

**Published:** 1893-10

**Authors:** Emil Schreier

**Affiliations:** Vienna, Austria


### THE TREATMENT OF INFECTED ROOT-CANALS WITH KALIUM AND NATRIUM.[1]

BY DR. EMIL SCHREIER, VIENNA, AUSTRIA.

I HAVE taken the liberty to request a place on the programme of this distinguished body for the reason that I entertain the hope that the subject of my paper is of such importance for daily practice that it cannot fail to arouse the interest of a large proportion of those present. It is my purpose to lay before you a procedure for the antiseptic treatment of infected root-canals, which from its great simplicity and ease of application, as well as on account of the many excellent results which have been obtained therewith, deserves consideration from this distinguished assembly. I refer to the method of treatment introduced by me with kalium-natrium (potassium and sodium).

For the sake of brevity I shall limit myself to developing the principles which underlie the method. Should I succeed in arousing the interest of the audience, discussion will undoubtedly take place, when I shall have the opportunity to express myself more at length upon this subject, which I have thoroughly elaborated. When a tooth with gangrenous or necrotic pulp comes under treatment, the dentist is confronted with the task of removing as far as possible a gelatinous, slightly consistent mass from a capillary tube, and this having been accomplished, to introduce into the same canal an antiseptic for purposes of disinfection. You are all aware how much time, patience, and skill are necessary for this operation. The average dentist has enough trouble in many cases in simply probing the canal with a delicate needle, not to speak of cleansing, and much less filling the same; he is, accordingly, compelled to leave out of consideration any thought of saving the tooth. Such cleansing, however, is unnecessary, if it be possible to convert the septic contents of the canal into an aseptic condition, and the operation is much simplified if it be possible to effect the transformation by the simple introduction of a nerve-needle.

My method seeks to fill the first indication by a chemical decomposition of the putrescent contents, in which the root-canal serves as a test-tube; the second indication is fulfilled in the development of a substance which is readily taken up by a nerve-needle, and suf-

---

[1] Read before the World's Columbian Dental Congress, Chicago, August, 1893.

ficiently adhesive for introduction into the canal. This substance which you here see consists of kalium and natrium in a metallic state. I pierce its paraffin stopper with a nerve-needle chosen at random. You observe a delicate deposit resembling quicksilver on the needle. I now dip the needle in a glass of water; the needle describes a fiery tract therein. In the root-canal in question there exists a putrescent mass. This consists of water and the decomposition product of albumen, the latter consisting especially of fats and fatty acids.

These substances have been formed by the influence of bacteria, and serve as a culture-medium for the various species contained therein. If I now introduce my preparation into the canal with the needle, decomposition of the watery contents will occur, with development of a considerable amount of heat. Potassium and sodium hydroxides are formed, which, in combination with the fat of the pulp, form soap. The characteristic gangrenous odor is accordingly changed into a well-marked soapy smell. A portion of the alkalies possess the well-known property of rendering albuminous substances soluble. Thus any remains of tissue adherent to the walls of the canal are dissolved, the latter become macerated, and access to the dentine canaliculi is possible sooner than can be effected by any other method thus far employed. Destruction of the organic contents of these canals is now possible. You will readily understand that in consequence of such destruction the disagreeable discoloration which too frequently occurs will be absent, and that the lime-salts of the tooth proper are in no wise injuriously affected by the treatment.

The introduction of the potassium and sodium has the additional effect of destroying the bacteria, partly by the heat produced, and partly by the new products formed. The contents of the canal have been transformed into a sterile and probably antiseptic mass. and thus the development of new colonies of bacteria is prevented. Everything has thus been accomplished which precedes permanent filling of the tooth.

A series of questions will no doubt spontaneously arise in your minds which will take form somewhat as follows: Have any particles of the septic contents of the canal been forced through the apical foramen before sterilization has been complete, and so caused infection of the alveoli? Has the destruction of the bacteria been shown to have been certainly accomplished? And finally, does the preparation adhere to the nerve-needle sufficiently to be easily transformed to the canal?

It will not be difficult to give a satisfactory answer to all these questions. I hope to have the opportunity of demonstrating my method on the living subject, and you will see how the transmitted mass travels in the direction of least resistance—that is, into the orifice of the canal next the pulp chamber—and wells up alongside of the needle. But the results of practice better than mere theoretical deductions demonstrate the groundlessness of such apprehensions. Reports from various sources are at hand as to the results of the preparation in practice. They are all eminently satisfactory. This would be impossible if the infection of the alveoli had occurred in any but the most insignificant proportion of cases. That the bacteria are actually destroyed I have proved by cultivation experiments, with the full description of which I shall not weary you. Hardly any one would seriously doubt the possibility of the method practised by me in effecting the destruction of organic life. I shall best succeed in convincing you that the preparation has sufficient consistency to adhere to the needle, by passing it around in actual contact with the needle. I shall probably have an opportunity of expressing myself upon various questions which may be raised in the discussion. It is scarcely necessary for me to state that my plan of treatment should only be practised with the coffer-dam. In an assembly like this, this fact will appear self-evident. Of course care must be exercised in manipulating with the preparation. With proper care the preparation is free from all danger.

A further question may be raised, whether the methods heretofore employed do not give satisfactory results—that the introduction of a new one is superfluous. I believe that I am entitled to say that the plan of treatment proposed by me is founded upon correct principles, and meets the obvious indications as regards ease and rapidity of application and certainty of result. It is, to say the least, the equal of any method. Every practitioner is in a position by the aid of my preparation to save with rapidity, ease, and well-nigh with certainty, teeth that have been seriously affected, and that without special preliminary preparation and without troublesome appliances. Thus the benefit of treatment of the root becomes possible for the masses. Inasmuch as the greater portion of mankind is forced to lose the teeth for lack of the means of calling in the aid of the dentist at the proper time, I assert that my method marks an important epoch in the progress of root-treatment, and I take the liberty of requesting you to submit the method proposed by me to your distinguished consideration.

